# Coping and Perception of Prognosis in Patients With Indolent Non-Hodgkin’s Lymphoma

**DOI:** 10.1093/oncolo/oyad295

**Published:** 2023-11-03

**Authors:** Richard A Newcomb, P Connor Johnson, Daniel Yang, Katherine Holmbeck, Joanna Choe, Anisa Nabily, Porsha Lark, Tejaswini Dhawale, Hermioni L Amonoo, Areej El-Jawahri

**Affiliations:** Division of Hematology and Oncology, Department of Medicine, Massachusetts General Hospital, Boston, MA, USA; Harvard Medical School, Boston, MA, USA; Division of Hematology and Oncology, Department of Medicine, Massachusetts General Hospital, Boston, MA, USA; Harvard Medical School, Boston, MA, USA; Duke University School of Medicine, Durham, NC, USA; Division of Hematology and Oncology, Department of Medicine, Massachusetts General Hospital, Boston, MA, USA; Division of Hematology and Oncology, Department of Medicine, Massachusetts General Hospital, Boston, MA, USA; Division of Hematology and Oncology, Department of Medicine, Massachusetts General Hospital, Boston, MA, USA; Division of Hematology and Oncology, Department of Medicine, Massachusetts General Hospital, Boston, MA, USA; Division of Hematology and Oncology, Department of Medicine, Massachusetts General Hospital, Boston, MA, USA; Harvard Medical School, Boston, MA, USA; Harvard Medical School, Boston, MA, USA; Department of Psychosocial Oncology and Palliative Care, Dana-Farber Cancer Institute, Boston, MA, USA; Division of Hematology and Oncology, Department of Medicine, Massachusetts General Hospital, Boston, MA, USA; Harvard Medical School, Boston, MA, USA

**Keywords:** indolent lymphomas, psychological distress, coping, prognostic awareness, coping with prognosis

## Abstract

**Background:**

Indolent non-Hodgkin’s lymphomas (iNHL) are a heterogenous group of mostly incurable diseases with prolonged illness courses and prognostic uncertainty. Yet, studies evaluating coping and perception of prognosis are limited.

**Methods:**

We conducted a cross-sectional study of adults newly diagnosed with iNHL in the past 3 months at a single academic center. We assessed quality of life (QOL: Functional Assessment of Cancer Therapy—General), psychological symptoms (Hospital Anxiety and Depression Scale), coping (Brief-COPE), and perception of prognosis (Prognosis Awareness Impact Scale).

**Results:**

We enrolled 70.6% (48/68) of eligible patients. Patients had older age (mean = 66.9,sd = 10.5), were female (60.4%), predominantly identified as White (85.4%), and had at least received a college degree (75%). Chronic lymphocytic leukemia (39.6%) and follicular lymphoma (33.3%) were the most common diagnoses. Overall, 27.1% and 14.6% of patients reported clinically significant anxiety and PTSD symptoms, respectively. Patients highly utilized acceptance (56.2%), seeking emotional support (47.9%), and denial (47.9%) as coping strategies at diagnosis. While 66.7% of patients recalled their oncologist assessment of illness as incurable, only 35.4% reported that the illness is unlikely to be cured. Overall, 45.8% indicated that they were worried about prognosis and 31.2% reported perseverating on their prognosis. Higher emotional coping with prognosis was associated with fewer anxiety (*B* = −0.6, *SE* = 0.2, *P* < .001), depression (*B* = −0.3, *SE* = .1, *P* = .005), and PTSD (*B* = −1.3, *SE* = 0.4, *P* < .001) symptoms and better QOL (*B* = 1.7, *SE* = 0.4, *P* < .001).

**Discussion:**

Patients with iNHL report substantial psychological distress, a diversity of coping strategies, and complex cognitive understanding of their prognosis. Interventions, which address prognostic uncertainty and promote positive emotional coping with prognosis, may ameliorate psychological distress in this population.

## Teaser

Indolent non-Hodgkin’s lymphomas are a heterogenous group of mostly incurable diseases with prolonged illness courses and prognostic uncertainty. This study evaluated coping strategies and perception of prognosis in this patient population.

## Graphical Abstract

**Graphical Abstract:**
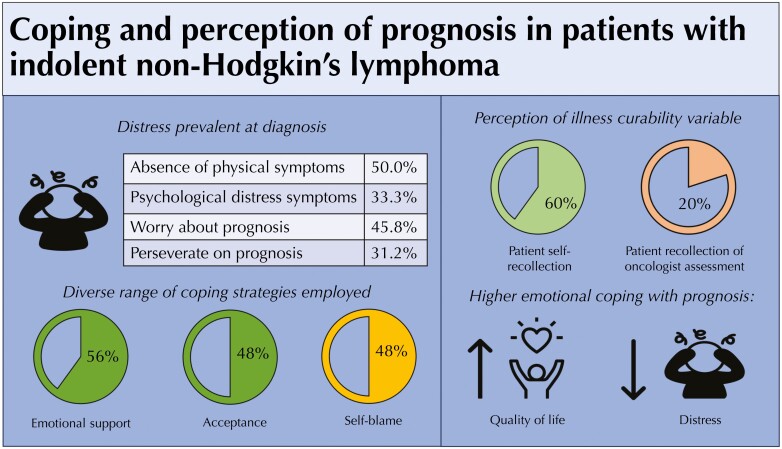

**Implications for Practice**

Patients newly diagnosed with indolent lymphoma frequently experience psychological distress, threatening perceptions of illness, and prognosis-related concerns, even in the absence of physical symptoms. Higher emotional coping with prognosis was associated with higher quality of life and less psychological distress at diagnosis. Oncology clinician awareness and recognition of prognosis-related concerns at diagnosis of indolent lymphoma are important. Supportive care interventions to promote higher emotional coping with prognosis have the potential to reduce psychological distress in this patient population.

## Background

Indolent non-Hodgkin lymphomas (iNHL) are a heterogenous group of chronic hematologic malignancies that are often incurable and have prolonged illness courses requiring sequential therapies with intermittent periods of remission and surveillance.^1,2^ While the majority of patients with iNHL carry favorable long-term prognoses, some patients with iNHL ultimately relapse, and a minority may die as a result of their illness.^3^ In addition, a subset of patients with iNHL will experience disease transformation to aggressive lymphomas, which require urgent chemotherapy, and clinicians cannot accurately predict which patients will go on to experience disease transformation. Accordingly, patient-centered prognostication at diagnosis and relapse remains an ongoing challenge due to lack of tailored prognostic assessment measures.^4,5^

Due to significant uncertainty around their illness trajectory, patients with iNHL often experience symptoms of anxiety.^6-8^ In the first year of illness, anxiety is prevalent occurring in 20%-30% of patients regardless of treatment strategy.^9^ At diagnosis, patients predominantly cite concerns about their lymphoma becoming more aggressive or requiring a change in treatment strategy.^9^ While some patients experience improvement in anxiety symptoms over time, others, particularly those receiving active surveillance suffer from persistent distress impacting functional, social, and emotional well-being.^9-11^ Interestingly, the impact of short-and long-term psychological distress on quality of life (QOL) is less clear. Some studies demonstrate decreased short-and long-term QOL when compared to the general population; however, others do not.^10-14^ Independent of illness effect on QOL, the diagnosis of iNHL exacts a persistent psychological toll. Long-term survivors of iNHL identify feelings of threat related to their illness years after diagnosis.^15^ However, unlike patients with aggressive hematologic malignancies, no studies have formally characterized the prevalence of post-traumatic stress disorder (PTSD) symptoms in patients with iNHL.^16,17^ Given patient concerns regarding disease transformation and need for future treatments, prognostic understanding may be intimately related to persistent feelings of threat related to iNHL. As diagnosis may be a formative time in processing prognosis, it is critical to assess the relationship between baseline prognostic understanding and psychological distress including PTSD symptoms in patients with iNHL.

Coping is critical to every aspect of the illness experience of patients with hematologic malignancies especially managing the psychological distress associated with diagnosis, treatment, surveillance, and survivorship.^18-20^ Patients may use a range of coping strategies to manage their illness experience which can be organized into a framework of approach-oriented and avoidant coping. Approach-oriented coping strategies are cognitive-behavioral strategies patients use to directly confront stressors that commonly accompany the illness experience.^21^ On the other hand, avoidant coping strategies involve withdrawing from the stress.^21^ Prior cross-sectional studies suggest patients with iNHL positively cope with their illness and simultaneously experience significant fear of cancer recurrence.^15,22,23^ Furthermore, in a randomized clinical trial comparing rituximab maintenance versus rituximab retreatment upon progression, patients using avoidant coping strategies, regardless of study arm experienced increased illness-related anxiety, general anxiety, and lower QOL over time.^24^ Thus, choice of coping strategy shapes longitudinal experience and outcomes in patients with iNHL. However, how patients with iNHL cope with prognostic uncertainty is not clear. It is conceivable that coping with prognostic uncertainty impacts psychological distress and QOL. Indeed, understanding the relationship between coping and prognostic uncertainty may be important to ameliorate psychological distress and to improve QOL in this patient population and merits further investigation.

Perception of prognosis is a multidimensional construct of prognostic awareness that incorporates cognitive understanding, emotional coping, and adaptive response (ie, the capacity to use prognostic awareness to inform life decisions).^25^ Perception of prognosis captures the intersection of illness perception, prognostic awareness, and coping and is associated with psychological distress, particularly symptoms of anxiety.^26^ Furthermore, in patients with aggressive hematologic malignancies, higher emotional coping and adaptive response to prognosis are associated with lower psychological distress and higher QOL.^27^ Thus, perception of prognosis in iNHL may inform the development of psychological distress and impact QOL. Yet, perception of prognosis has not been previously described in patients with iNHL. As initial impressions of illness are formed at diagnosis, it is particularly important to characterize baseline perception of prognosis in this patient population.

Thus, the time of diagnosis may critically inform development of psychological distress including PTSD symptoms, use of coping strategies, and most importantly perception of prognosis in patients with iNHL. Given the formative importance of diagnosis, we conducted a cross-sectional analysis of baseline data from a longitudinal prospective study of patients newly diagnosed with iNHL. In this study, we principally aim to characterize baseline perceptions of illness, use of coping strategies, and perception of prognosis. We secondarily aim to evaluate the association of perception of prognosis at diagnosis with baseline psychological distress and QOL.

## Methods

### Study Design

We conducted cross-sectional analyses of baseline data of patients with iNHL from a longitudinal prospective cohort study of patients newly diagnosed with indolent hematologic malignancies (iNHL, myeloproliferative neoplasm, and myelodysplastic syndrome) at Massachusetts General Hospital, Boston, MA, from August 2021 to July 2022. For the iNHL cohort, we screened the lymphoma clinic for potentially eligible patients. With permission from their oncologist, we approached patients within 3 months of diagnosis with iNHL. Prior to analyses of baseline data, we had completed enrollment of the iNHL study cohort.

### Participants

In the iNHL cohort, eligible participants were adults (≥18 years of age) diagnosed with iNHL in the past 3 months. We defined iNHL as a diagnosis of indolent B-cell or T-cell lymphomas/leukemias including chronic lymphocytic leukemia/small lymphocytic lymphoma (CLL/SLL), classic follicular lymphoma, marginal zone lymphoma, hairy cell leukemia, indolent B-cell lymphoma not otherwise specified, cutaneous T-cell lymphoma, large granular lymphocytic leukemia, lymphoplasmacytic lymphoma, or Waldenström’s macroglobulinemia. Eligibility criteria also entailed the ability to read and respond to questions and complete questionnaires in English or willingness to complete questionnaires with assistance from an interpreter. We excluded patients with psychiatric or cognitive conditions which the oncologist believed would preclude the ability to provide informed consent. Within 2 weeks of study consent, patients completed the baseline assessments and were registered.

### Sociodemographic and Clinical Data

At enrollment, patients self-reported demographic information including age, gender, education, race, ethnicity, marital status, employment status, and income. We also collected clinical variables such as diagnosis, stage at diagnosis, treatment strategy, and treatment intent (ie, curable, incurable) based on the chemotherapy intent ordering system and clinician notes from the electronic health record.

### Patient-Reported Measures

#### Psychological Distress

We used the 14-item Hospital Anxiety and Depression Scale (HADS) to assess depression and anxiety symptoms.^28^ The HADS contains two 7-item subscales that measure symptoms of depression and anxiety within the past week. Each subscale ranges from 0 to 21, and higher subscale scores indicate worse distress symptoms. Scores greater than 7 indicate clinically significant symptoms. We used the PTSD Checklist-Civilian Version (PCL-C) to assess PTSD disorder symptoms.^29^ The PCL-C is a 17-item self-reported measure that evaluates the severity of PTSD symptoms. Scores range from 17 to 85 with higher scores indicating worse PTSD symptoms. Scores greater than 29 are considered clinically significant.

#### Quality of Life

We assessed patient QOL with the Functional Assessment of Cancer Therapy-General (FACT-G), which measures physical, functional, emotional, and social well-being during the past week. Scores range from 0 to 108 with higher scores representing better QOL.^30^ FACT-G is a validated QOL measure in patients with iNHL.^31^

#### Illness Perception

We measured patients’ perception of their illness using the 8-item Brief Illness Perception Questionnaire (BIPQ).^32,33^ Items include consequences (“How does your illness affect your life?”), timeline (“How long do you think your illness will continue?”), personal control (“How much control do you feel you have over your illness?”), treatment control (“How much do you think your treatment can help your illness?”), identity (“How much do you experience symptoms from your illness?”), concern (“How concerned are you about your illness?”), illness coherence (“How well do you feel you understand your illness?”), and emotional representation (“How much does your illness affect you emotionally?”). Each item is rated on a 0-10 scale. Total score (0-80) is calculated by summing the scores of each item. Higher scores indicate more threatening illness perception.

#### Coping

Patients’ use of coping strategies was measured with the Brief COPE, a 28-item questionnaire that assesses the use of 14 coping methods with 2 items for each method.^34^ To reduce questionnaire burden for participants, we limited our assessment to 8 coping strategies previously used in other studies to assess coping in patients with hematologic malignancies: use of active coping, positive reframing, acceptance, emotional support, religious coping, self-blame, denial, and behavioral disengagement.^18,35^ Scores for each scale range from 2 to 8, and higher scores indicate greater use of that coping strategy.

#### Perception of Prognosis

We measured patients’ perception of their prognosis using the Prognostic Awareness Impact Scale (PAIS), a novel tool to measure prognostic awareness as a multidimensional construct undergoing validation and with prior use in patients with hematologic malignancies.^25,27,36^ The PAIS is a 34-item questionnaire that measures 3 domains: cognitive understanding of prognosis, emotional coping with prognosis, and adaptive response (ie, the capacity to use prognostic awareness to inform life decisions).^25^ The cognitive understanding domain consists of 2 items including a question asking if their oncologist had said their cancer was curable (“Yes,” “No,” “My oncologist has not said”) and a question asking patients to report the likelihood that they would be cured of their cancer, assessed on a 7-point scale. As in prior studies of patients with incurable solid and hematologic malignancies, we dichotomized responses into “Incurable” (defined as “no chance—0% chance of cure” and “very unlikely—less than 10% chance of cure” vs “Curable” (defined as all other responses).^36-39^ We, additionally, created a dichotomous variable to capture concordance in patient self-report of prognosis and patient recollection of oncologist prognostic assessment. The emotional coping domains consists of 8 items (score range of 0-24) with higher scores indicating better emotional coping with prognosis. The adaptive response domain consists of 12 items (score range: 0-36) with higher scores indicating better adaptive response to prognosis.

### Statistical Analysis

We calculated descriptive statistics, including means or medians for continuous variables and proportions for categorical variables. As in previous studies,^18-20,35,40^ we used the median split method to describe the distribution of coping domains in our sample because there are no validated Brief-COPE cutoffs for high or low coping. We calculated the median scores for each of the 7 coping domains, then described the proportion of patients with a score greater than the median as “high utilizers” for each coping strategy. Patients, whose score was the median or lower, were included in the “low utilizers” group. We considered the median score (8) as the cutoff point for “high utilizers” for the emotional support coping strategy use because the median scores were the same as the highest possible score (8).

Given limited sample size, we used unadjusted linear regression models to examine associations of patient perception of prognosis with patient-reported outcomes (QOL and symptoms of anxiety, depression and PTSD). We used complete case analyses without accounting for missing data. With a low missingness rate of 1.1%, there were no observed differences in missing data by coping. We considered a 2-sided *P*-value < .05 as statistically significant. We performed all statistical analyses using R version 4.2.1 (R Foundation for Statistical Computing, Vienna, Austria).

## Results

### Patient Characteristics

Between September 2021 and September 2022, we enrolled 70.6% (48/68) eligible patients newly diagnosed with iNHL. Table 1 displays baseline sociodemographic and clinical characteristics. Mean age was 66.9 years (SD = 10.5). The majority of patients were female (60.4%), self-identified as White (85.4%), had at least received a college degree (75%), and were married or living with a partner (81.2%). One patient (2.1%) required the assistance of an interpreter (Vietnamese). The most common diagnoses were chronic lymphocytic leukemia/small lymphocytic lymphoma (39.6%) and follicular lymphoma (33.3%). Most patients underwent upfront active surveillance (62.5%). A minority had limited stage disease treated with curative intent radiation therapy (12.5%).

**Table 1.** Sociodemographic and clinical characteristics.

	*n* (%)
Age, mean (SD)	66.9 (10.5)
Female	29 (60.4)
Race	
White	41 (85.4)
African-American	1 (2.1)
Asian	4 (8.3)
American Indian	1 (2.1)
Middle Eastern	1 (2.1)
Hispanic ethnicity	0 (0.0)
English as primary language	47 (97.9)
Relationship	
Married or cohabitating	36 (75.0)
Non-cohabitating relationship	3 (6.2)
Single, never married	2 (4.2)
Divorced/separated	2 (4.2)
Widowed	5 (10.4)
Live alone	8 (16.7)
Highest education level	
High school degree or equivalent	2 (4.2)
Some college or associate degree	9 (18.8)
College degree	9 (18.8)
Some graduate education	7 (14.6)
Advanced graduate degree	20 (41.7)
Missing	1 (2.1)
Employment	
Work (full or part time)	19 (39.6)
Retired	23 (47.9)
Missing	6 (12.5)
Diagnosis	
CLL/SLL	19 (39.6)
Follicular lymphoma	16 (33.3)
Marginal zone lymphoma	10 (20.8)
Other	3 (6.2)
Radiation therapy	7 (14.6)
Management strategy	
Active surveillance	31 (64.6)
Curative intent treatment	6 (12.5)
Palliative intent treatment: time limited	10 (20.8)
Palliative intent treatment: indefinite	1 (2.1)

Abbreviations: CLL: chronic lymphocytic leukemia; SLL: small lymphocytic lymphoma; MALT: mucosal associated lymphoid tissue.

### Psychological Distress and QOL at Diagnosis

At the time of diagnosis, a minority of patients reported clinically significant symptoms of depression (6.3%, 3/48), anxiety (27.1%, 13/48), and PTSD (14.6%, 7/48). Overall, one-third (16/48) reported any clinically significant symptom of psychological distress. The mean overall baseline QOL was 87 (interquartile range [IQR]: 80.3-96.2). There were no statistically or clinically significant differences in psychological distress or QOL between patients undergoing active surveillance and those receiving cancer-directed therapy. There were no differences in distress or QOL between patients receiving curative intent radiation therapy and those undergoing surveillance or palliative intent therapy.

### Illness Perception and Coping Strategies at Diagnosis

**Figure 1.**
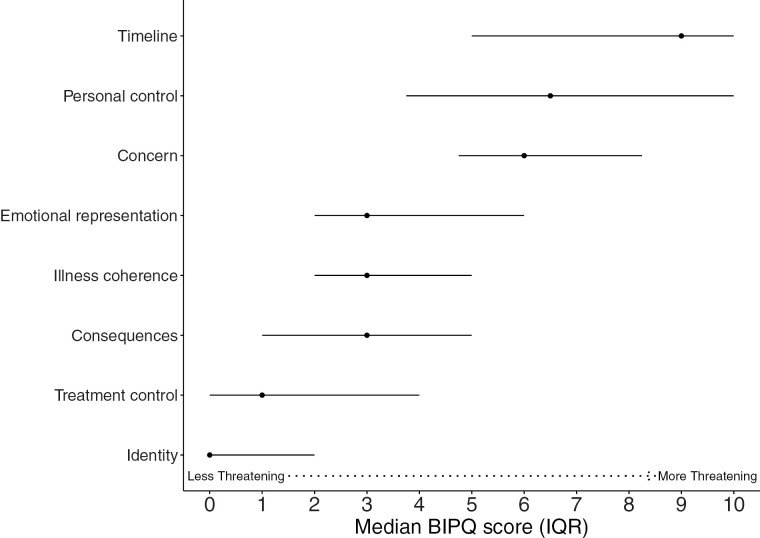
Median (interquartile range 25th percentile-75th percentile) plots of illness perception domains as measured by Brief Illness Perception Questionnaire (BIPQ) at diagnosis of indolent non-Hodgkin’s lymphoma. Higher scores represent higher levels of threat related to illness.

Fig. 1 displays median scores of BIPQ illness perception domains. Patients reported higher levels of threat related to timeline (median = 9, IQR = 5-10), concern (median = 6, IQR = 4.75-8.25), and loss of personal control (median = 6.5, IQR = 3.75-10). On the other hand, patients perceived their illness as less threatening related to illness coherence (median = 3, IQR = 2-5), emotional representation (median = 3, IQR = 2-6), treatment control (median = 1, IQR = 0-4), and consequences median = 3, IQR = 1-5). Most patients reported no physical symptoms related to their iNHL (median = 0, IQR = 0-2). Most patients highly used acceptance (56.2%), seeking emotional support (47.9%), and denial (47.9%) as coping strategies. A significant minority of patients highly used self-blame (22.9%) and behavioral disengagement (22.9%).

### Perception of Prognosis at Diagnosis

**Figure 2.**
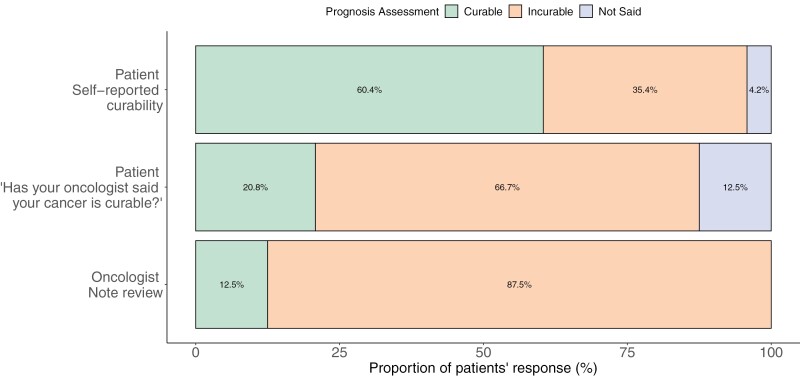
Cognitive understanding of prognosis: Comparison of prognosis assessments among patient self-report, patient recollection of oncologist’s report, and documentation of oncologist’s prognosis in the electronic health record.

**Figure 3.**
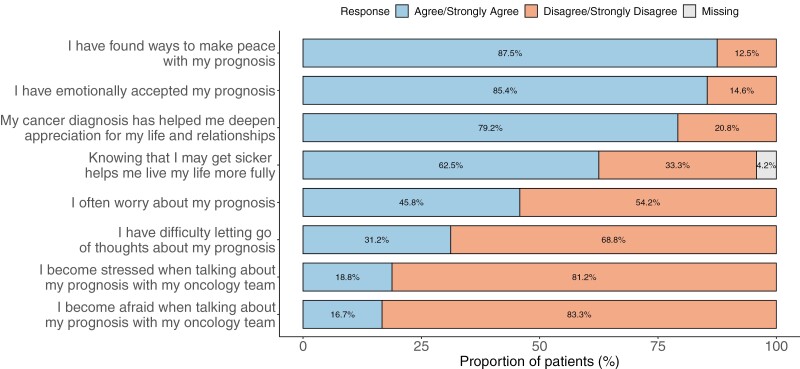
Emotional coping with prognosis: Proportion of patients who “agree or strongly agree” and “disagree or strongly disagree” with statements reflecting emotional coping with prognosis of indolent non-Hodgkin’s lymphoma.

Fig. 2 displays cognitive understanding of prognosis among patient self-report (“How likely are you to be cured of your cancer?”), patient recollection of oncologist’s report (“Has your oncologist said that your cancer is curable?”), and documentation of oncologist’s prognosis in the EHR. While 66.7% of patients stated that their oncologist told them that their illness is incurable, only 35.4% reported that the illness is unlikely to be cured. Most oncologists’ (87.5%) identified their patients’ iNHL as “Incurable.” The median overall score of emotional coping with prognosis was 16 [IQR: 13.7-18.2]. Fig. 3 shows responses to the PAIS emotional coping with prognosis items. While 45.8% communicated “often worrying about prognosis,” 31.2% reported “difficulty letting go of thoughts of prognosis.” A subset of patients (16.7%-18.8%) shared feelings of fear and/or stress when talking about prognosis with their oncology teams. On the other hand, most patients relayed that they had emotionally accepted (85.4%) and found ways to make peace (87.5%) with their prognosis. The median overall score of adaptive coping with prognosis was 24 [IQR: 22-27.3]. Most patients exhibited adaptive responses to prognosis including feeling grateful for each day (79.2%) and discovering self-strength (70.8%). In addition, most patients believed that knowing prognosis was helpful for making decisions about treatment (93.8%), preparing for the future (91.7%), maintaining hope (93.7%), coping with the disease (93.7%), and focusing on meaningful activities (75.0%). Interestingly, a significant minority of patients found that knowledge of prognosis was not helpful to enhance relationships with loved ones (35.4%) nor develop a greater sense of closeness with others (37.5%).

### Association Between Perception of Prognosis and Psychological Distress and QOL

Table 2 displays the association between domains of perception of prognosis and symptoms of psychological distress and QOL. Emotional coping with prognosis was associated with fewer baseline symptoms of anxiety (*B* = −0.6, *SE* = 0.2, *P* < .001), depression (*B* = −0.3, *SE* = 0.1, *P* = .005), and PTSD (*B* = −1.3, *SE* = 0.4, *P* < .001) and higher baseline QOL (1.7, *SE* = 0.4, *P* < .001). Cognitive understanding and adaptive response were not associated with baseline psychological distress and QOL.

**Table 2.** Univariate association of perception of prognosis domains and patient-reported outcomes including symptoms of psychological distress (anxiety, depression, and post-traumatic stress disorder [PTSD]) and quality of life.

Perception of prognosis domain	Anxiety		Depression		PTSD		Quality of life	
	B (SE)	*P*	B (SE)	*P*	B (SE)	*P*	B (SE)	*P*
Cognitive concordance	0.6 (1.1)	.59	0.7 (0.8)	.34	3.1 (2.6)	.25	−0.4 (3.0)	.90
Emotional coping	−0.6 (0.2)	<.001	−0.3 (0.1)	.005	−1.3 (0.4)	<.001	1.7 (0.4)	<.001
Adaptive coping	−0.1 (0.1)	.61	−0.1 (0.1)	.35	−0.3 (0.3)	.35	0.57 (0.3)	.08

## Discussion

In this cross-sectional study of patients newly diagnosed with iNHL, we found a substantial minority of patients experience symptoms of psychological distress, most notably anxiety and PTSD. In addition, patients express high levels of concerns about their illness and perceive the indefinite illness timeline and lack of personal control over illness as particularly threatening. At diagnosis, patients with iNHL express hope for illness curability, and the majority simultaneously report understanding that their oncologist has said their illness is incurable. While patients worry about their prognosis, many report positive emotional coping strategies and correspondingly implemented adaptive life behaviors. However, more than one-third did not believe their illness experience enhanced relationships with family and loved ones. Finally, higher emotional coping with prognosis was strongly associated with decreased baseline psychological distress and improved QOL.

Our study reports a substantial burden of psychological distress in patients diagnosed with iNHL and newly describes clinically significant PTSD symptoms in a significant minority. Importantly, curative intent of treatment and choice of active surveillance as upfront management were not associated with psychological distress. Prior studies have similarly reported a high prevalence of anxiety and depression symptoms, independent of treatment strategy.^7,9^ It is important for lymphoma clinicians to be aware of the psychological impact of iNHL on all patients at diagnosis. The finding of clinically significant baseline PTSD symptoms is particularly striking. Patients with acute myeloid leukemia and those undergoing hematopoietic stem cell transplantation also suffer from PTSD symptoms.^16,17^ In patients with aggressive hematologic malignancies, PTSD symptoms are often linked to immense physical symptoms and psychological distress from treatment with high-dose chemotherapy. Yet, in our population of iNHL, many patients had no physical symptoms, underwent active surveillance, and still report trauma symptoms. Our findings suggest the diagnosis of iNHL may be traumatic and its psychological toll pernicious and durable. Indeed, perceptions of illness threat previously described in long-term survivors with iNHL are notably similar to the PTSD symptoms in our newly diagnosed population.^15^ Future longitudinal investigations into the persistence and significance of PTSD symptoms are needed.

Our study additionally details perception of illness and use of coping strategies in iNHL. Patients identified their iNHL as threatening related to illness chronicity, lack of personal control, and high levels of illness-specific concern. In patients receiving treatment for chronic lymphocytic leukemia, Arrato and colleagues recently identified that threatening perceptions of physical symptoms, consequences, and concern were highly associated with mood symptoms and cancer associated stress.^41^ In our population predominantly undergoing active surveillance, we observed a high prevalence of psychological distress despite absence of physical symptoms and perception of threatening illness consequences. Future studies should focus on longitudinal characterization of illness perception and its association with psychological distress, particularly in patients undergoing active surveillance. Furthermore, it is notable that patients newly diagnosed with iNHL employ a diversity of coping strategies. While the majority use approach-oriented coping strategies such as acceptance and seeking emotional support from others, a significant minority highly utilize avoidant strategies including denial and self-blame. It is possible that threatening perception of illness may augment avoidant coping strategy use and should be investigated in future longitudinal studies. Prior studies similarly highlight the importance of identifying avoidant coping in patients with iNHL.^9^ Our study demonstrates that diagnosis may be an ideal time to develop interventions to address threatening illness perception and to mitigate avoidant coping. Interventions incorporating psychologists or social workers, who have expertise in promoting adaptive coping, could be considered in future supportive care interventions.^42^

To our knowledge, our study is among the first to detail perception of prognosis in patients newly diagnosed with iNHL. Our findings indicate that many patients with iNHL exhibit incongruent cognitive understanding of prognosis. Despite accurately reporting their oncologist’s perception of prognosis as “Incurable,” most patients did not personally identify their illness as “Incurable.” A similar phenomenon is observed in patients with advanced solid malignancies.^26^ Patients with advanced lung cancer notably “swing” between a more and less realistic understanding of their cancer, and over time integration of prognosis-specific hopes and worries is integral to effective engagement in shared-decision making.^43^ Patients with iNHL may share a similar cognitive process, and future investigation is warranted. In our study, cognitive understanding of prognosis was notably not associated with increased baseline psychological distress nor decreased quality of life. Nevertheless, larger studies and longitudinal data are needed to determine the significance of cognitive understanding of prognosis to psychological distress, QOL, and medical decision making for patients with indolent hematologic malignancies.

In addition, we observed that emotional coping with prognosis was strongly associated with baseline psychological distress and QOL. In our experience, patients with iNHL, particularly those on active surveillance, are casually described as the “worried well” suggesting their distress is unwarranted. However, our findings suggest their distress is measurable and more importantly potentially modifiable. First, higher emotional coping with prognosis was associated with less baseline psychological distress and higher QOL. While many patients intrinsically adopted healthy coping behaviors after their diagnosis, a significant minority reported a high degree of concern about their prognosis. Supportive care interventions to provide high quality disease education and to promote healthy coping behaviors at the time of diagnosis could ameliorate distress associated with perception of prognosis and should be rigorously developed and tested. Second, a significant number of patients did not believe their iNHL diagnosis brought them closer to loved ones. Studies have previously demonstrated a need for augmented social support for patients with iNHL, and our findings reaffirm this unmet need.^10,11^ Group interventions in chronic illness such as chronic graft-versus-host have demonstrated promising efficacy to improve QOL and reduce psychological distress.^44^ Accordingly, future studies should focus on development of group interventions to augment social support for patients with iNHL.

The current analysis has several limitations. First, the study cohort was derived from a single academic center that serves patient populations with limited diversity. Race, ethnicity, culture, and socioeconomic status may impact perception of prognosis and use of coping strategies, and our findings may not be generalizable to broader patient groups. Similarly, our study population was predominantly female and married. Gender and marital status may influence perception of prognosis and coping and thus may impact generalizability of study findings. Future studies to evaluate coping and prognostic uncertainty in more diverse patient populations with iNHL are needed. Second, small sample size may also impact generalizability. Third, the cross-sectional design limits applicability across the illness course. Fourth, our study occurred during the COVID-19 pandemic, and some patients with iNHL were at higher risk of severe viral infection and decreased likelihood of adequate immune response to vaccination.^45,46^ Timing of our study could have contributed to increased psychological distress related to the COVID-19 pandemic. Finally, it is worth highlighting prognosis in patients with iNHL is complex, as intensive therapies such as allogeneic transplant or adoptive cellular therapy are potentially curative. Discussion of these therapies could have contributed to patient’s cognitive understanding of their prognosis.

## Conclusion

Patients newly diagnosed with iNHL report psychological distress, threatening perceptions of illness, complex cognitive understanding of prognosis, and prognosis-related concerns. In addition, patients used a diversity of coping strategies and implemented adaptive behaviors in response to their prognosis. Interventions to address prognostic uncertainty and to promote positive emotional coping with prognosis in patients with iNHL are an unmet need and have the potential to ameliorate psychological distress and improve QOL in this patient population.

## Data Availability

The data underlying this article will be shared on reasonable request to the corresponding author.
